# Pooled prevalence and factors associated with insecticide-treated net use among pregnant women in malaria high-burden countries in sub-Saharan Africa: a multilevel mixed-effects analysis

**DOI:** 10.1186/s41182-025-00855-w

**Published:** 2025-11-19

**Authors:** Edson Mwebesa, Benson Musinguzi, Ismail D. Legason, Robert Opoke, Bosco B. Agaba, Rornald Muhumuza Kananura, Ann Mwangi

**Affiliations:** 1 Department of Mathematics, Faculty of Science, Muni University, Arua, Uganda; 2 School of Science and Aerospace Studies, Moi University, Eldoret, Kenya; 3 Department of Medical Laboratory Sciences, Faculty of Health Sciences, Muni University, Arua, Uganda; 4 Department of Immunology and Molecular Biology, School of Biomedical Sciences, College of Health Sciences, Makerere University, Kampala, Uganda; 5 Department of Public Health, Faculty of Health Sciences, Muni University, Arua, Uganda; 6 Department of Biology, Faculty of Science, Muni University, Arua, Uganda; 7 Faculty of Medicine, Mbarara University of Science and Technology, Mbarara, Uganda; 8 National Malaria Control Division, Ministry of Health, Kampala, Uganda; 9 Center of Excellence for Maternal and Newborn Health, Makerere University School of Public Health, Kampala, Uganda; 10 Demographic Dynamic and Population Health Unit, African Population and Health Research Center, Dakar, Senegal

**Keywords:** Pregnant women, Malaria, Insecticide-treated nets, Sub-Saharan Africa

## Abstract

**Introduction:**

Malaria remains a significant public health challenge among pregnant women in sub-Saharan Africa (SSA). Proper use of insecticide-treated nets (ITNs) is a key intervention for malaria; however, their utilisation remains low and suboptimal in high-burden countries. Investigation of correlates of use of ITNs among pregnant women in only high-burden malaria countries SSA are dearth. This study aimed to identify the pooled prevalence and factors associated with ITNs use among pregnant women in seven malaria high-burden countries in SSA.

**Methods:**

Secondary analysis of recent (2017–2021) Malaria Indicator Survey (MIS) data from seven malaria high-burden countries in SSA was utilised. Women aged 15–49 years, who were pregnant during the survey, were included in this study, resulting in a pooled sample size of 4950 women. Pooled prevalence of ITN use was obtained through the use of proportions, with a 95% confidence interval (CI). A multilevel mixed-effect logistic regression model was run to obtain factors associated with ITNs use among pregnant women at 5% level of significance. Data analysis was done in Stata v17.0 and R 4.5.0.

**Findings:**

The pooled prevalence of ITNs use among these countries was 63.8 [95% CI 61.8, 65.9], low in Ghana (49.2%) and high in Niger (90.5%). Having a primary level of education [aOR = 1.36, 95% CI 1.21, 1.53] compared to those with no formal education, having given birth to one to two children [aOR = 1.24, 95% CI 1.06, 1.44] compared to those with no births yet were associated with higher odds of ITN use. 14% of the total variation in ITN use was attributable to differences between countries [intra-cluster correlation (ICC) = 0.14, 95% CI 0.06, 0.20] and 32% of the variation in ITN use within countries is attributed to differences between regions [ICC = 0.32, 95% CI 0.21, 0.46].

**Conclusions:**

The prevalence of ITN use was suboptimal, and socio-demographic and household factors are associated with ITN use among pregnant women, with substantial in-country variation underscoring the role of regional context in ITN utilization. These findings suggest that beyond individual and household determinants, local and regional contexts play a critical role in shaping ITN usage patterns. Interventions should, therefore, be tailored not only to socio-demographic profiles but also to regional and local disparities in access, awareness, and implementation effectiveness.

## Introduction

Malaria remains one of the most significant public health challenges globally, with sub-Saharan Africa (SSA) bearing the heaviest burden. According to the World Health Organisation (WHO) World Malaria Report 2024, an estimated 263 million malaria cases were reported globally in 2023, with SSA accounting for 95% of all cases and 96% of malaria-related deaths [1]. Pregnant women are among the most affected groups due to both biological and social vulnerabilities. While biological factors such as immune modulation and placental parasite sequestration increase susceptibility to infection and malaria complications [2–4], social determinants play a critical role in exposing pregnant women to malaria. For example, limited access to preventive interventions, such as insecticide-treated nets and intermittent preventive treatment [5], gender inequality in decision-making [6], and low socioeconomic status [7] can reduce women’s ability to protect themselves effectively. In rural and low-income settings, poor housing conditions, inadequate access to antenatal care, and dependence on subsistence livelihoods further heighten exposure risks [8]. In addition, women’s caregiving and domestic responsibilities often increase their time outdoors during peak mosquito biting hours [9–11], compounding their risk of infection. Malaria during pregnancy (MiP) is associated with significant adverse outcomes, including maternal anaemia, placental malaria, intrauterine growth restriction, low birth weight, preterm delivery, and increased neonatal mortality [12, 13]. These effects not only threaten maternal and child health but also strain healthcare systems and contribute to the cycle of poverty in households and the affected regions [14, 15].

One of the most effective and widely promoted strategies for preventing malaria in pregnancy is the use of insecticide-treated nets (ITNs) [16, 17], which creates a physical barrier against malaria-carrying mosquitoes, protecting both the mother and the unborn child from infection. ITNs have been shown to significantly reduce malaria transmission, morbidity, and mortality, particularly when used consistently and correctly [17, 18]. WHO recommends that all individuals living in malaria-endemic areas, including pregnant women, sleep under ITNs every night [19]. Despite widespread campaigns and large-scale distribution programs across SSA, coverage and actual utilisation of ITNs remain suboptimal in many high-malaria burden settings [20].

Several studies have explored factors influencing ITN ownership and use among pregnant women, identifying determinants, such as education level, socio-economic status, household composition, number of children, and urban versus rural residence [21–23]. For instance, multi-country studies such as Terefe et al. (2023) [24] and Demoze et al. (2024) [25] examined ITN use in East African countries, while Ameyaw (2021) [8] investigated use of ITNs among pregnant women in 21 SSA countries, including low-burden malaria countries like Rwanda. While these studies provide valuable insights, their scope include diverse malaria burden contexts, which may dilute findings relevant to high-burden settings, where malaria remains a leading cause of morbidity and mortality among pregnant women. Furthermore, existing research frequently lacks comparative analyses that account for regional and contextual disparities, such as variations in health system infrastructure or cultural practices, limiting the applicability of findings to tailored malaria control strategies in high burden countries. he current study addresses this gap by focusing exclusively on malaria high-burden countries that adopt country-led malaria control initiatives guided by World Health Organisation (WHO) [26]. This focus is critical, because these countries face unique challenges, including strained health systems and high disease prevalence, requiring targeted, context-specific interventions. By emphasizing country-led approaches, this study aligns with WHO’s Global Malaria Programme, which prioritizes national ownership and adaptation of strategies to local epidemiology and resources, thereby offering insights that can enhance the effectiveness of malaria control policies in the most affected regions.

**Fig. 1:**
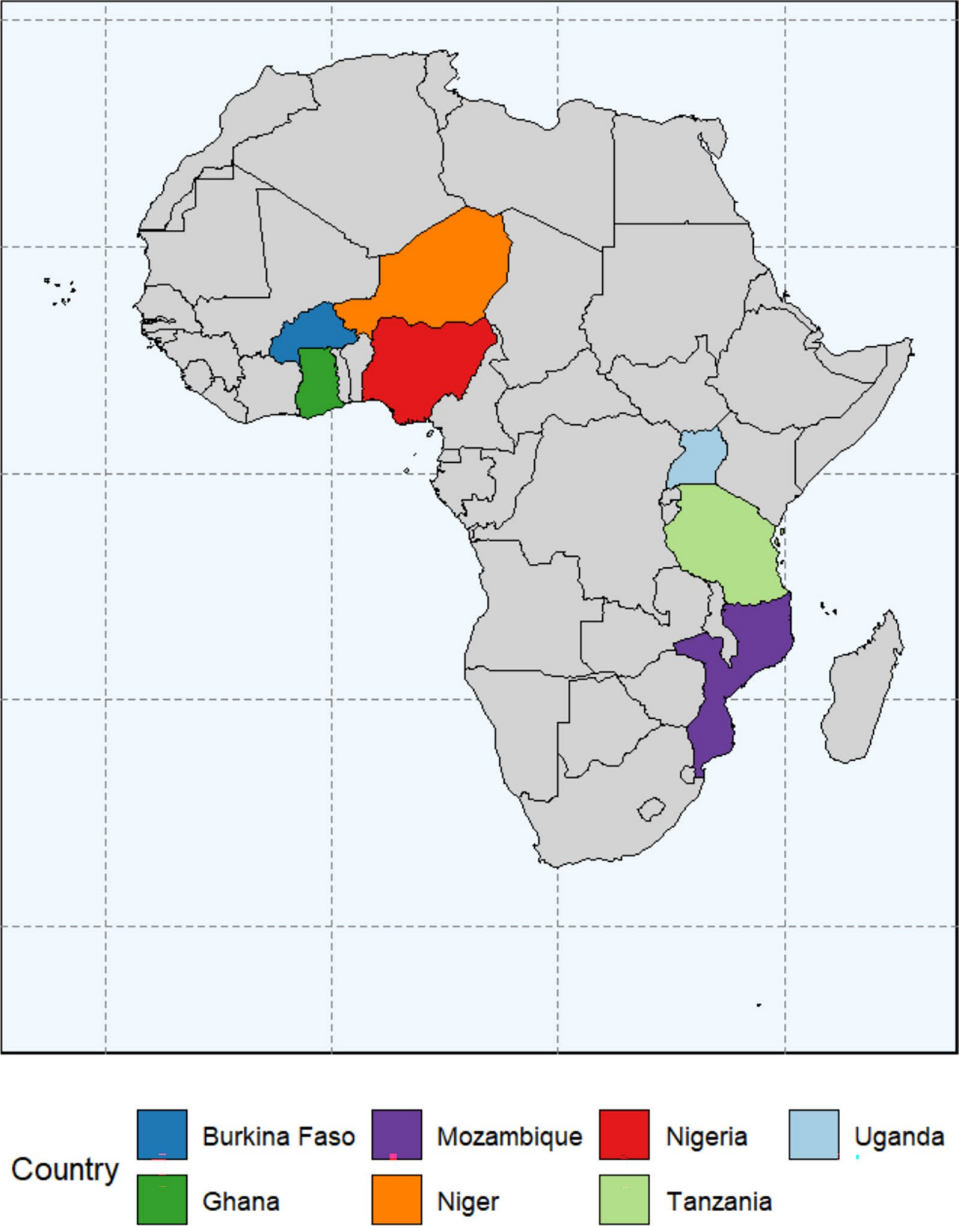
Map of selected African countries included in the study

Recognising the continued burden of malaria in specific countries, the WHO launched the High Burden to High Impact (HBHI) initiative in 2018 to drive intensified efforts in 11 countries that collectively account for over 70% of the global malaria burden [26]. Seven of these countries are in SSA: Nigeria, Burkina Faso, Ghana, Mozambique, Niger, Uganda, and Tanzania (Fig. 1). These countries share commonalities in disease burden but vary in geography, health infrastructure, and socio-cultural factors influencing health behaviours. Understanding ITN use among pregnant women in these HBHI countries is thus essential for informing targeted interventions and optimising the impact of malaria prevention programs. These countries share a common model for malaria control program, that focuses on strengthening political will, improve strategic information to guide action, and implement better policies. One of the many programs implemented in these countries is distribution of ITN especially in vulnerable populations, such as pregnant women through periodic mass distribution campaigns, distribution at health facilities during antenatal and immunisation visits, community outreach programs, and provisions for sale on open market, in shops, private hospitals and pharmacies [27–32].

Despite strategic importance of ITN use, there is limited comparative and pooled evidence on the prevalence and predictors of ITN use among pregnant women across HBHI countries in SSA. Previous studies have largely focused on individual countries and have not adequately addressed broader regional patterns or contextual influences [8, 33, 34]. This gap in the literature constrains efforts to develop integrated and scalable approaches for improving ITN use, where it is needed most. This current study aimed to fill this gap by examining the pooled prevalence of ITN use among pregnant women in seven high-burden countries in sub-Saharan Africa, identify factors associated with utilisation and examine the extent of variations in ITN use among pregnant women between and within countries. Combining data from these countries in a pooled analysis enhances understanding of ITN use and generalizability across SSA by increasing sample size for greater statistical power, and providing a more robust basis for developing targeted interventions and national policies to improve ITN utilization. This allows for more reliable conclusions than single country studies, reveals country disparities in ITN use, and informs tailored strategies for malaria elimination. By leveraging recent Demographic and Health Survey (DHS) data and applying multilevel analytic methods, the study provides critical insights to inform targeted and equitable malaria prevention strategies across the region.

### Data source and study population

The study utilized recent malaria indicator surveys (MIS) from seven high burden to high impact malaria countries, that is, Burkina Faso (November 2017–March 2018), Ghana (September–November 2019), Nigeria (2021), Niger (August–October 2021), Mozambique (March–July 2018), Tanzania (2017) and Uganda (2018–19) (Fig. 1). These surveys were conducted by the country’s National Bureau of Statistics (NBS) with the support of the International Classification of Functioning, Disability and Health (ICF) through the Demographic and Health Survey (DHS) Program [35]. These countries were selected, because they are high burden to high impact countries, availability of data and representation of sub-Saharan Africa (SSA), where Eastern (Uganda, Tanzania), Western (Burkina Faso, Ghana, Nigeria, Niger), and Southern (Mozambique) (Fig. 1). Two-stage stratified cluster sampling was applied to select clusters (enumeration areas), after which households were selected. Women aged 15–49 years, within selected households, were selected to be part of the sample. The MIS surveys are part of the Demographic Health Survey (DHS) program that follows standard methods using the women, household and biomarkers questionnaires. Detailed survey procedures for the DHS are found elsewhere, for example [36–39]. Pregnant non-refugee women during the survey were included in this study, resulting in a pooled sample size of 4950 (unweighted) women and 4971 (weighted). Pregnant refugee women were excluded from this study, because such data existed only in Uganda, and we contended that it would not be representative enough if included in the study.

The MIS data used in this study were from seven countries, collected from 2017 up to 2021. The seven countries represent three SSA regions, East Africa, West Africa and South Africa. The weighted sample was used to identify the pooled prevalence and factors associated with ITN use among pregnant women. The largest sample size was obtained from Nigeria, 1264 (25.4%), while the least was from Ghana, 353 (7.1%). The highest prevalence of ITN use was 90.5% in Niger, while Ghana had the lowest ITN use, 49.2%. The pooled prevalence was 63.8% [95% CI 61.8%, 65.9%]. The regional ITN use showed that in Western Africa (Burkina Faso, Ghana, Nigeria, Niger) was 61.6% [95% CI 59.1, 64.0], Eastern Africa (Uganda and Tanzania) was 61.7% [95% CI 57.4, 65.9], while Southern Africa (Mozambique) was 83.2% [95% CI 78.7, 86.9]. Results are shown in Table 1.

**Table 1:** Country sample size and prevalence

Country	Years	Unweighted	Weighted	Weighted prevalence
		Count (%)	Count (%)	%[95% CI]
Burkina Faso	2017–18	622 (12.6)	604 (12.1)	59.4 [54.8, 63.9]
Ghana	2019	364 (7.4)	353 (7.1)	49.2 [43.2, 55.2]
Mozambique	2018	482 (9.7)	510 (10.3)	83.2 [78.7, 86.9]
Nigeria	2021	1192 (24.1)	1264 (25.4)	50.3 [46.6, 54.0]
Niger	2021	662 (13.4)	688 (13.8)	90.5 [87.8, 92.7]
Tanzania	2017	942 (19.0)	896 (18.0)	57.4 [51.9, 62.7]
Uganda	2018–19	686 (13.9)	656 (13.2)	67.5 [61.0, 73.4]
Pooled sample size		4950	4971	
Pooled prevalence				63.8 [61.8, 65.9]
Regional prevalence				
Western Africa				61.6 [59.1, 64.0]
Eastern Africa				61.7 [57.4, 65.9]
Southern Africa				83.2 [78.7, 86.9]

Source: MIS data

### Study variables

The study outcome was ITN use defined as respondent having slept under mosquito bed net the previous night (Yes/No). The predictor variables included age group of the woman (15–19, 20–24, 25–29, 30–34, and 35–49), education level of the woman (No education, primary, secondary, higher), total number of children ever born (no births, 1–2 births, 3–5 births, 6 or more births), births in the last 3 years (none, 1–3 births), number of children 5 years and under in household (0, 1, 2, 3, 3 or more), sex of household head (male, female), number of household members (1–3, 4–6, 7 or more), wealth index (poorest, poorer, middle, richer, richest), type of toilet facility (improved, unimproved), source of drinking water (improved, unimproved), type of place of residence (urban, rural) and country (Burkina Faso, Ghana, Mozambique, Nigeria, Niger, Tanzania, Uganda).

### Data analysis

Data analysis was done in three stages. The description of sample characteristics was conducted using proportions and their 95% confidence intervals. This approach was used to estimate the pooled proportion of women sleeping under insecticide-treated nets, which is the primary outcome of the study. The study predictors analyzed by proportions and their 95% confidence intervals included age, education level, parity, number of births in the last 3 years, number of children under five in the household, sex of the household head, number of household members, combined wealth index, type of toilet facility, source of drinking water, type of residence, and country. Cross-tabulations were used to examine the distribution of mosquito net use with these predictors. A multilevel mixed-effects logistic regression model was fitted for all characteristics, except for country. A binary logistic multilevel mixed-effects model was fit, because the units of analysis (women) are nested within geographical regions (specified sub regions in a given country), and these regions were nested within countries. As a result, observations within clusters are dependent and thus traditional logistic regression model, which assume independence of observations, would fail to account for varying effects at different levels. Multilevel modeling identifies and models group level variations and allows for assessment of individual and contextual level factors simultaneously and offers precise estimates [40]. The null model (Model 0) was fitted to check whether insecticide-treated net use varied across grouping variables. The second model (Model 1) contained woman-level factors. The third model (Model 2) included household-level factors. The final model (Model 3) contained factors in Model 1, Model 2 and type of place of residence to identify which ones were associated with mosquito net use. The mixed effect model used in this study is of the form:

$$\text{logit}\left[P\left({{\varvec{Y}}}_{ijk}=1\right)\right]= {\mathbf{X}}_{ijk}{\varvec{\beta}}+ {\mathbf{Z}}_{ijk}{\mathbf{u}}_{j}+ {\mathbf{W}}_{ijk}{\mathbf{v}}_{{\varvec{k}}}$$

where ${{\varvec{Y}}}_{ijk}$ is ITN use (No = 0, Yes = 1) for woman $i$ in region $j$, within a country $k$, $\text{logit}\left[P\left({{\varvec{Y}}}_{ijk}=1\right)\right]$ is the log-odds of ITN use for a woman $i$, ${\mathbf{X}}_{ijk}$ is a vector of fixed-effect predictors for woman $i$ in region $j$, within a country $k$, ${\varvec{\beta}}$ is a vector of fixed-effect coefficients of X. ${\mathbf{Z}}_{ijk}$ is the vector related to random effects for the region, ${\mathbf{W}}_{ijk}$ is the vector of random effects for country. ${\mathbf{u}}_{j}$ is the vector of random intercepts associated with the $j$ th level of region, while ${\mathbf{v}}_{{\varvec{k}}}$ is the vector of random intercepts associated with $k$ th level of the country. ${\mathbf{u}}_{j}$ and ${\mathbf{v}}_{j}$ are assumed to be normally distributed with mean 0 and variance–covariance matrices ${\Sigma }_{u}$ and ${\Sigma }_{v}$, respectively.

Factors were considered significant if the *p* value ≤ 0.05. Odds Ratios (OR) and their 95% confidence intervals (CIs) were presented. The inter-cluster correlation coefficient (ICC) for each model was presented. The Akaike information criterion (AIC) was used for model comparison. Stata Version 17.0 and R 4.5.0 were utilized in the analysis. Survey weights were adjusted for in analysis of the data to cater for unequal probabilities of selection, non-response bias, for post-stratification adjustments and obtain valid population inference [41, 42].

## Results

This section presents the pooled prevalence of mosquito bed net use in Nigeria, Burkina Faso, Ghana, Mozambique, Niger, Uganda, and Tanzania (malaria high-burden countries) in SSA, sample characteristics (woman level, household level, residence and country), the distribution of these characteristics by insecticide-treated nets (ITNs) status and factors associated with the use of ITNs.

### Socio-demographic characteristics and pooled prevalence of ITN use of pregnant women based on recent MIS data

Among the pregnant women in this study, most of them were aged 20–24 years [26.9, 95% CI [25.4, 28.4], with no formal education [38.5, 95% CI 36.2, 40.8], who had given birth to 1–2 children [33.8, 95% CI 32.2, 35.5]. Most women had not had birth 3 years preceding the survey [60.9, 95% CI 59.0, 62.7], lived in households with one child [35.1, 95% CI 33.3, 37.0], headed by male [88.5, 95% CI 87.3, 89.6] with 4–6 members [37.8, 95% CI 36.0, 39.6]. Most women came from poorer households [22.0, 95% CI 20.3, 23.8], had unimproved toilet facilities [56.1, 95% CI 53.6, 58.5], and mainly used improved water sources [66.4, 95% CI 63.8, 68.8]. Most women were from rural areas [75.3, 95% CI 73.0, 77.4], and from Nigeria [25.4, 95% CI 23.2, 27.7%]. The pooled prevalence of ITN use among pregnant women in HBHI countries in SSA was 63.8% [95% CI 61.8, 65.9]. Of pregnant women who slept under the bed net, most were those aged 20–24 years [28.0, 95% CI 26.2, 30.0] with no formal education [38.7, 95% CI 35.9, 41.5], had given birth to one to two children [34.6, 95% CI 32.6, 36.7] but had not given birth in the last 3 years [59.8, 95% CI 57.5, 62.1].

In addition, most of these women lived in households with one child [35.5, 95% CI 33.3, 37.7], in male headed households [89.5, 95% CI 88.0, 90.8], with 4 to 6 household members [38.8, 955 CI 36.6, 40.9], with poorer wealth index [22.9, 95% 20.8, 25.1] and unimproved toilet facility [57.8, 95% CI 54.9, 60.7]. Significant associations between ITN use and woman’s education level (*p* < 0.001), total number of children ever born (*p* = 0.002), number of children 5 and under in household (*p* = 0.007), sex of household head (*p* = 0.0337), number of household members (*p* < 0.001), type of toilet facility (*p* = 0.019) and country (*p* < 0.001) were observed. Results are shown in Table 2.

**Table 2:** Sample distribution of currently pregnant women in seven malaria high-burden to high-impact countries in sub-Saharan Africa based on recent MIS data

	Overall	Slept under the bed net		*p* value
		No	Yes	
	Percent [95% CI]	Percent [95% CI]	Percent [95% CI]	
		36.2 [34.1, 38.2]	63.8 [61.8, 65.9]	
Age groups [Recoded]				
15–19	16.7 [15.4, 18.0]	17.0 [15.0, 19.2]	16.5 [15.0, 18.1]	0.232
20–24	26.9 [25.4, 28.4]	24.8 [22.4, 27.4]	28.0 [26.2, 30.0]	
25–29	26.0 [24.5, 27.6]	26.8 [24.3, 29.6]	25.5 [23.7, 27.5]	
30–34	16.6 [15.3, 17.9]	17.8 [15.8, 20.1]	15.9 [14.4, 17.4]	
35–49	13.9 [12.8, 15.2]	13.6 [11.7, 15.7]	14.1 [12.7, 15.7]	
Highest educational level				
No Education	38.5 [36.2, 40.8]	38.1 [35.0, 41.3]	38.7 [35.9, 41.5]	< 0.001
Primary	34.7 [32.6, 36.9]	30.3 [27.1, 33.8]	37.2 [34.7, 39.8]	
Secondary	23.2 [21.5, 25.0]	26.0 [23.4, 28.7]	21.6 [19.6, 23.8]	
Higher	3.6 [3.0, 4.4]	5.6 [4.4, 7.2]	2.5 [1.9, 3.3]	
Total children ever born [Recoded]				
No births	23.5 [21.9, 25.2]	26.9 [24.2, 29.8]	21.6 [19.8, 23.5]	0.002
1–2 births	33.8 [32.2, 35.5]	32.5 [29.9, 35.3]	34.6 [32.6, 36.7]	
3–5 births	29.2 [27.7, 30.7]	29.2 [26.7, 31.9]	29.1 [27.4, 31.0]	
6 or more births	13.5 [12.3, 14.8]	11.4 [9.5, 13.5]	14.7 [13.2, 16.3]	
Births in last 3 years [Recoded]				
None	60.9 [59.0, 62.7]	62.7 [59.9, 65.4]	59.8 [57.5, 62.1]	0.096
1–3 Births	39.2 [37.3, 41.0]	37.3 [34.6, 40.1]	40.2 [37.9, 42.5]	
Number of children 5 and under in household [Recoded]				
0 Children	28.5 [26.8, 30.2]	29.0 [26.6, 31.6]	28.1 [26.1, 30.3]	0.007
1 Child	35.1 [33.3, 37.0]	34.4 [31.5, 37.6]	35.5 [33.3, 37.7]	
2 Children	23.7 [22.1, 25.3]	21.3 [19.1, 23.8]	25.0 [23.1, 27.0]	
3 or more	12.8 [11.5, 14.2]	15.2 [12.9, 17.8]	11.4 [10.0, 13.0]	
Sex of household head				
Male	88.5 [87.3, 89.6]	86.9 [84.7, 88.8]	89.5 [88.0, 90.8]	0.034
Female	11.5 [10.4, 12.7]	13.1 [11.3, 15.3]	10.5 [9.2, 12.0]	
Number of household members [Recoded]				
1–3	26.3 [24.8, 27.9]	23.1 [20.8, 25.7]	28.1 [26.2, 30.1]	< 0.001
4–6	37.8 [36.0, 39.6]	36.1 [33.1, 39.2]	38.8 [36.6, 40.9]	
7 or more	35.9 [34.0, 37.9]	40.8 [37.3, 44.3]	33.1 [31.0, 35.3]	
Wealth index combined				
Poorest	21.0 [19.2, 22.9]	22.4 [19.8, 25.3]	20.2 [18.2, 22.4]	0.119
Poorer	22.0 [20.3, 23.8]	20.5 [17.9, 23.4]	22.9 [20.8, 25.1]	
Middle	21.2 [19.6, 23.0]	19.8 [17.4, 22.4]	22.0 [20.0, 24.3]	
Richer	19.2 [17.5, 21.0]	18.9 [16.5, 21.7]	19.3 [17.3, 21.5]	
Richest	16.6 [14.8, 18.6]	18.4 [15.8, 21.2]	15.6 [13.5, 18.0]	
Type of toilet facility				
Improved	43.9 [41.5, 46.4]	47.1 [43.6, 50.7]	42.2 [39.3, 45.1]	0.019
Not Improved	56.1 [53.6, 58.5]	52.9 [49.3, 56.4]	57.8 [54.9, 60.7]	
Source of drinking water				
Improved	66.4 [63.8, 68.8]	68.3 [64.8, 71.6]	65.2 [62.2, 68.2]	0.133
Not Improved	33.7 [31.2, 36.2]	31.7 [28.4, 35.2]	34.8 [31.9, 37.8]	
Type of place of residence				
Urban	24.7 [22.6, 27.0]	26.2 [23.2, 29.4]	23.9 [21.4, 26.7]	0.205
Rural	75.3 [73.0, 77.4]	73.8 [70.6, 76.8]	76.1 [73.3, 78.7]	
Country				
Burkina Faso	12.2 [10.4, 14.1]	13.6 [11.3, 16.4]	11.3 [9.5, 13.4]	< 0.001
Ghana	7.1 [5.9, 8.5]	10.0 [8.1, 12.2]	5.5 [4.4, 6.9]	
Mozambique	10.3 [8.4, 12.4]	4.8 [3.5, 6.4]	13.4 [11.0, 16.2]	
Nigeria	25.4 [23.2, 27.7]	34.9 [31.4, 38.6]	20.1 [17.8, 22.5]	
Niger	13.8 [11.7, 16.3]	3.6 [2.7, 4.8]	19.6 [16.6, 23.1]	
Tanzania	18.0 [15.9, 20.4]	21.2 [17.6, 25.3]	16.2 [14.1, 18.6]	
Uganda	13.2 [11.2, 15.5]	11.9 [9.1, 15.3]	14.0 [11.8, 16.5]	

Source: MIS data

### Prevalence of ITN use among pregnant women in seven malaria-high-burden countries in sub-Saharan Africa based on recent MIS data

**Fig. 2:**
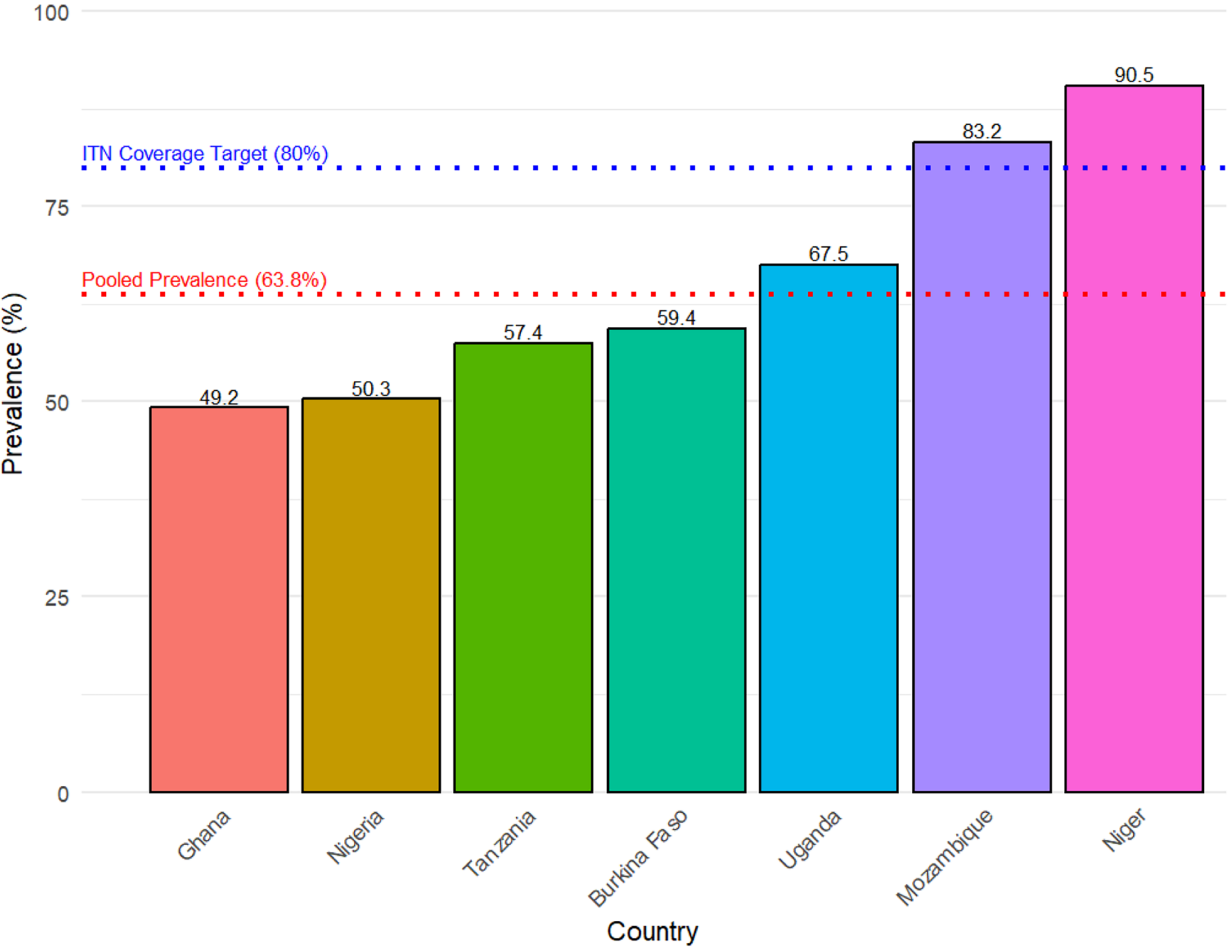
Prevalence of ITN use among pregnant women in malaria-high-burden to high-impact countries in SSA. Source: MIS data

The results reveal that the prevalence of ITN use among pregnant women was 59.4% in Burkina Faso, 49.2% in Ghana, 83.2% in Mozambique, 50.3% in Nigeria, 90.5% in Niger, 57.4% in Tanzania and 67.5% in Uganda. Results are shown in Fig. 2. The pooled prevalence of ITN use among these countries was 63.8 [95% CI 61.8, 65.9]. Results are shown in Table 2.

### Factors associated with insecticide-treated net use among pregnant women in malaria-high burden countries in sub-Saharan Africa

The factors associated with insecticide-treated net use were investigated using a multivariable multilevel mixed-effects logistic regression model to account for the hierarchical structure of the data, where pregnant women nested within regions, nested within countries and allowed for the examination of multiple factors simultaneously while controlling for potential confounding variables. The results revealed that, those with primary education were more likely to use ITN [aOR = 1.36, 95% CI 1.21, 1.53] compared to pregnant women with no education, while those with secondary education were more likely to use ITN compared to those with no education [aOR = 1.35, 95% CI 1.14, 1.61]. Women with one to two births, compared to those with no births, were more likely to use ITN [aOR = 1.24, 95% CI 1.06, 1.44]. Having children of 5 years and below in the household was positively associated with the use of ITN. In particular, those living with two children [aOR = 1.32, 95% CI 1.10, 1.57] compared to households with no children. Living in households headed by females was negatively associated with use of ITN among pregnant women in SSA [aOR = 0.74, 95% CI 0.58, 0.95] compared to households headed by males. The results also revealed that having more than four members living in households was negatively associated with ITN use, with [aOR = 0.67, 95% CI 0.55, 0.81] for households with 4–6 members, and [aOR = 0.38, 95% CI 0.29, 0.50] for households with 7 or more members compared to having 1–3 members in a household.

The random effects part of the model quantifies the variability in ITN use at country and regional levels in each country, which is not explained by covariates included in the model. Based on the empty model (Model 0), the variation in ITN use between countries was 0.55 (SE = 0.25), while the variation in ITN use between regions within countries was 0.74 (SE = 0.74). The country's intra-cluster correlation (ICC) was [ICC = 0.12, 95% CI 0.05, 0.24], suggesting that 12% of the total variation in ITN use among pregnant women was attributable to differences between countries. The region | country ICC [ICC = 0.28, 95% CI 0.19, 0.40], showing that 28% of variations in ITN within countries are attributed to differences between regions. This shows that there exists substantial clustering of ITN use at both regional and country levels. The Akaike Information Criterion (AIC) for the null model was 5691.41. From Model 4 (final model), the results revealed that the variation in ITN use between countries was 0.67 (SE = 0.32), while the variation in ITN use between regions within countries was 0.87 (SE = 0.20). The country’s intra-cluster cluster correlation (ICC) was [ICC = 0.14, 95% CI 0.06, 0.28], suggesting that 14% of total variation in ITN use among pregnant women was attributable to differences between countries after controlling for covariates. This might be explained by differences in country-level factors such as national policies, governance, healthcare systems and economic conditions that influence the use of ITN. The region within country ICC was [ICC = 0.32, 95% CI 0.21, 0.46], showing that 32% of variations in ITN use within countries is attributed to differences between regions in the same country. This higher proportion of variations suggests that regional level covariates are more influential in explaining variation in ITN use than country level factors. This implies that where a pregnant woman lives in terms of region strongly predicts her ITN use. In other words, a grouping of country and regions (within a country) accounts for larger variation in ITN use than country alone. This shows that there exists substantial clustering of ITN use at both regional and country levels after controlling for different factors. The ITN use is not solely dependent on individual level characteristics as variability in ITN use is explained by the country and more so, regions within these countries. The Akaike Information Criterion (AIC) for the final model was 5389.48, lower than all the other models, suggesting that it was a better fit. Results are presented in Table 3.

**Table 3:** Multivariable multilevel mixed effects logistic regression model for factors associated with insecticide-treated net use among pregnant women in malaria-high burden countries in sub-Saharan Africa

Covariates	Model 0 [Empty]	Model 1 aOR[95%CI]	Model 2 aOR[95%CI]	Model 3 aOR[95%CI]
Age groups [Recoded]				
15–19		Reference		Reference
20–24		1.18 [0.84,1.66]		1.16 [0.80,1.68]
25–29		1.10 [0.64,1.89]		1.17 [0.70,1.97]
30–34		1.06 [0.58,1.94]		1.11 [0.65,1.92]
35–49		1.19 [0.66,2.15]		1.31 [0.78,2.18]
Highest educational level				
No Education		Reference		Reference
Primary		1.34 [1.09,1.65]^**^		1.36 [1.21,1.53]^**^
Secondary		1.39 [1.05,1.84]^*^		1.35 [1.14,1.61]^**^
Higher		0.96 [0.60,1.52]		0.97 [0.67,1.40]
Total children ever born [Recoded]				
No births		Reference		Reference
1–2 births		1.31 [1.09,1.58]^**^		1.24 [1.06,1.44]^**^
3–5 births		1.23 [0.87,1.76]		1.35 [0.96,1.92]
6 or more births		1.23 [0.73,2.06]		1.62 [0.95,2.77]
Births in last 3 years [Recoded]				
None		Reference		Reference
1–3 Births		0.98 [0.82,1.17]		0.98 [0.78,1.23]
Number of children 5 and under in household [Recoded]				
0 Children			Reference	Reference
1 Child			1.19 [0.86,1.64]	1.07 [0.83,1.38]
2 Children			1.43 [1.12,1.82]^**^	1.32 [1.10,1.57]^**^
3 or more			1.14 [0.76,1.70]	1.07 [0.79,1.46]
Sex of household head				
Male			Reference	Reference
Female			0.73 [0.57, 0.94]^*^	0.74 [0.58, 0.95]^*^
Number of household members [Recoded]				
1–3			Reference	Reference
4–6			0.72 [0.56,0.92]^*^	0.67 [0.55,0.81]^**^
7 or more			0.43 [0.30,0.62]^**^	0.38 [0.29,0.50]^**^
Wealth index combined				
Poorest			Reference	Reference
Poorer			1.22 [0.86,1.75]	1.21 [0.83,1.76]
Middle			1.24 [0.88,1.75]	1.20 [0.85,1.71]
Richer			1.17 [0.65,2.09]	1.13 [0.63,2.01]
Richest			0.92 [0.45,1.90]	0.88 [0.49,1.58]
Type of toilet facility [Recoded]				
Improved			Reference	Reference
Not Improved			0.84 [0.69,1.01]	0.84 [0.71,0.99]^*^
Source of drinking water [Recoded]				
Improved			Reference	Reference
Not Improved			1.05 [0.89,1.25]	1.08 [0.92,1.27]
Type of place of residence				
Urban				Reference
Rural				0.96 [0.69,1.33]
Random effect				
Country-level variance [SE]	0.55 [0.25]	0.57 [0.25]	0.65 [0.31]	0.67 [0.32]
Country > Regional Level Variance [SE]	0.74 [0.74]	0.76 [0.16]	0.85 [0.19]	0.87 [0.20]
Country ICC [95% CI]	0.12 [0.05, 0.24]	0.12 [0.09, 0.24]	0.14 [0.06, 0.28]	0.14 [0.06, 0.28]
Region | Country ICC [95% CI]	0.28 [0.19, 0.40]	0.29 [0.19, 0.41]	0.31 [0.20, 0.45]	0.32 [0.21, 0.46]
Model fit statistics				
Log-likelihood	− 2842.71	− 2828.13	− 2706.91	− 2688.74
AIC	5691.41	5668.26	5425.81	5389.48
N	4950	4950	4830	4830

*aOR *adjusted odds ratio, *SE *standard error, *ICC* inter-cluster correlation coefficient, *AIC* Akaike information criterion, *CI* confidence interval

Source: MIS data.

* *p* < 0.05, ** *p* < 0.01

## Discussion

In this study, we found out that the seven countries had differing prevalence on ITN use among pregnant women with a 59.4% in Burkina Faso, 49.2% in Ghana, 83.2% in Mozambique, 50.3% in Nigeria, 90.5% in Niger, 57.4% in Tanzania, 67.5% in Uganda and with a pooled prevalence of 63.8% [95% CI 61.8–65.9]. Similar prevalence has been observed in Sierra Leone[43], although it is lower than that observed in 21 SSA countries [8]. However, higher ITN use has been observed in East Africa [24, 25], in Rwanda [44], North West Ethiopia [45], in Eastern Uganda, [46] and in a tertiary hospital in Nigeria [47]. Similar country-specific prevalence have been observed in Uganda [8] and Ghana [8, 48]. The World Health Organisation recommends universal coverage of ITNs and their use among pregnant women [49]; however, findings from this study reveal that ITN use is below the recommended global targets of 80% ITN use among pregnant women [50, 51]. Sub-optimal use of ITNs increases the risk of exposure to infective mosquito bites and may explain the high prevalence of malaria in Uganda [52], in Tanzania [53], in Nigeria [54], in Ghana [55], Burkina Faso [56], in Niger [57] and Mozambique [58]. The observed country-level disparities in ITN use among pregnant women, with highest in Niger and lowest in Ghana, reflect differences in health system capacity, distribution strategies, and socio-cultural contexts across Sub-Saharan Africa. Higher uptake of ITN likely results from differentiated malaria prevention programs. For example, frequent mass distribution campaigns, integration of ITN provision within antenatal care services, and strong community engagement is strongly observed in many SSA countries. On the other hand, lower utilization, for example, in Ghana and other countries may be linked to inconsistent campaign reach and cultural perceptions that discourage consistent net use, including discomfort during hot weather and beliefs about net cleanliness [59–62].

Close to 46% of the variation was explained by the differences between the countries and within the countries, with within the countries explaining the largest (32%). As such the individual-level variation (~ 54%) might be due to household income, education, or household structure. Understanding these factors could explain the differing utilisation of ITN and probably support country-specific interventions to improve ITN use. The intra-cluster correlations show variations in ITN use attributable to differences in countries and larger variation between regions within countries. This implies that although national malaria control policies and distribution campaigns influence overall uptake in ITNs, substantial heterogeneity persists at the subnational level. Similar findings across Sub-Saharan Africa suggest that regional and district disparities contribute significantly to uneven coverage and utilization [61, 63, 64]. The relatively high regional-level ICC underscores the need for geographically targeted interventions, ensuring equitable distribution of ITN, social and behavioral change communication, and health system strengthening at local levels [65, 66]. In the context of this study, policies should complement national malaria strategies with tailored regional efforts towards ITN uptake to address within-country inequalities and improve well-being of pregnant women. The observed regional variation in ITN use among pregnant women highlights the need for tailored malaria prevention strategies to local contexts instead of relying on uniform national-level approaches, and adopting national level policies that are contextualized to the national needs and malaria prevention demands. Understanding these contextual differences can guide more equitable allocation of limited resources, and strengthen health system responsiveness to local barriers and enablers of ITN use. Targeted, data-driven interventions that consider contextual, cultural, behavioral, and infrastructural factors are, therefore, critical to achieving universal ITN coverage among pregnant women across sub-Saharan Africa.

This study found that the education level of women (primary or secondary) was positively associated with sleeping under an ITN in the seven high-burden malaria countries in SSA. These findings are similar to those observed in sub-Saharan Africa [8], East Africa [24], Ghana, [67] and Rwanda [44]. However, some studies do not agree with these findings. Studies by Ankomah et al. (2012) and Adedokun et al. (2020) [34, 68] in Nigeria, Awunyo et al., (2025), Dun-Dery et al. (2022) and Klu et al. (2022) in Ghana [48] [22, 67] observed that the education of the woman was not significantly associated with ITN use among pregnant women. Despite this differing observations in these studies, educated pregnant women are more likely to use ITN as they are more likely to be aware of dangers of malaria in pregnancy, attend antenatal care, have the knowledge of proper ITN use and have greater autonomy and decision making power related to health needs and preventive practices than their counterparts [17, 24, 69].

Our findings observed a positive association between parity (one or two children) and ITN utilisation among pregnant women in malaria high-burden countries. This aligns with observations from Sierra Leone [43], where similar findings were noted among women who had given birth to five or more children, and in Ghana [22], where an association was found among women who had given birth to one or more children. However, the relations between ITN utilisation and parity were not observed in Rwanda [44], suggesting differences in contextual factors that influence ITN use. Furthermore, the study revealed a negative association between the use of ITN and the sex of the household being female, specifically where pregnant women resided. This implies a lower likelihood of ITN use in female-headed households compared to those in male-headed households. This finding is a direct parallel with observations in a multi-country study in SSA, where pregnant women in male-headed households were more likely to use ITN compared to female-headed households [8]. However, similar findings were observed in Sierra Leone [43]. This may be attributed to several interconnected socio-economic challenges often faced by female-headed households, such as greater economic vulnerability due to limited access to productive resources [70, 71], especially in low- and medium-income countries, and this impacts households’ ability to afford health commodities such as ITN. These financial constraints can directly impact a household's ability to afford essential health commodities and services like ITNs, even at a subsidised price or freely distributed.

This study also revealed that having children in the household was positively associated with ITN use among pregnant women, and having four or more household members in the house was negatively associated with ITN use among pregnant women. These findings suggest a complex interplay between household factors and preventive health behaviours, particularly in resource-limited households. Concerning household size, similar findings were observed in a multi-country study in SSA [24]. The positive relationship between the presence of children and ITN use among pregnant women may be due to awareness of the risks posed by malaria, particularly in under-fives. Therefore, mothers are more likely to embrace preventive measures. Similar patterns have been observed, where households with young children exhibit higher ITN utilisation due to increased health-seeking behaviour [72, 73]. On the other hand, the inverse relationship between household size and ITN use among pregnant women may indicate potential issues with the availability and allocation of ITNs. However, as this study was based on secondary data, these covariates need to be evaluated in primary studies.

The findings from this study reveals that there is a need for country specific and regional specific (regions within a country) malaria prevention strategies. This is evidenced by both country and regional variations in ITN use. The study observed high utilisation of ITN among educated women. This shows that there is need for tailoring ITN distribution and education efforts to women with lower education to improve the uptake of ITN among these women. Among other strategies to improve the uptake of ITN, integration of outreach programs, in addition to routine antenatal care would play a big role in improving ITN use.

This study utilised nationally representative MIS data conducted from 2017 to 2021 from these countries. This enhances credibility and comparability across countries as similar designs were applied during data collections and development of analytical plans. In addition, the study utilized a multilevel modelling approach which appropriately accounts for clustering and thus handles better dependencies within clusters and lead to more reliable estimates than traditional regression models. However, the study analysed data collected from 2017 to 2021. There is a possibility that more recent surveys may have been conducted in these countries, potentially reporting new data on the variables under investigation. However, this being population-based data sets, the study’s validity remains high with potential to inform countries to design strategies for improving optimal use of ITNs. The MIS has a large number of variables collected; however, this study analysed limited indicators related to ITN use and may be contextually different. In addition, the pooled analysis might have led to a loss in country-specific nuance and heterogeneity in program implementation. This was averted and minimised by the use of multilevel analysis to cater for group-level variations. Whereas the study observed factors associated with ITN use in seven countries in SSA, other high-malaria-endemic countries are not included in this study. The study proposes that future studies could seek to investigate these factors in all highly endemic countries in SSA. Future studies could also use qualitative data to obtain an in-depth understanding of contextual factors. The study examines factors associated with the use of ITN, but does not examine causal pathways on how these factors impact the uptake of ITN. Longitudinal studies would better capture ITN use dynamics which are not captured in cross-sectional studies. Unmeasured confounders, such as those related to cultural contexts, may influence use but are not included.

The study revealed that at least six out of every ten pregnant women used ITNs, although country-level prevalence varied. While this is encouraging, a notable gap remains in the utilisation of ITN if progress towards the elimination of malaria is to be achieved. The ITN use remains suboptimal and varies significantly within and between countries in this study. This highlights the need for further targeted interventions due to substantial in-country and between-country variations, underscoring the role of regional context in ITN utilization. Beyond individual and household determinants, local and regional contexts play a critical role in shaping ITN usage patterns.

Furthermore, higher educational attainment among women, having ever given birth to 1–2 children, and having two children under the age of five in the household were positively associated with ITN use. Conversely, female-headed households with one or more residents were negatively associated with the use of ITNs. These findings highlight important demographic and household characteristics that influence the use of ITNs among pregnant women, underscoring the need for targeted malaria prevention efforts. Interventions should, therefore, be tailored not only to socio-demographic profiles but also to regional disparities in access, awareness, and implementation effectiveness. Expanding ITN access especially among women with lower education level and those living in female-headed households, improving maternal health education, and strengthening regional monitoring could improve ITN uptake.

## Data Availability

The datasets analysed during the current study were obtained from MEASURE DHS program. Data can be obtained at: https://dhsprogram.com/data/dataset_admin/index.cfm

## Abbreviations

AIC: Akaike information criterion
aOR: Adjusted odds ratio
CI: Confidence interval
DHS: Demographic and health survey
ICC: Inter-cluster correlation coefficient
ICF: International classification of functioning, disability and health
ITN: Insecticide-treated net
ITNs: Insecticide-treated nets
MIS: Malaria indicator survey
NBS: National bureau of statistics
SE: Standard error
SSA: Sub-Saharan Africa
WHO: World Health Organization

## References

[1] WHO. World malaria report. 2023. https://www.wipo.int/amc/en/mediation/%0Ahttps://www.who.int/teams/global-malaria-programme/reports/world-malaria-report-2023. Accessed 1 Aug 2025

[2] Schantz-Dunn J, Nour NM. Malaria and pregnancy: a global health perspective. Rev Obstet Gynecol. 2009;2(3):186–92. 19826576 PMC2760896

[3] Obeagu EI, Obeagu GU. Protecting maternal health: strategies against HIV and malaria in pregnancy. Medicine. 2024;103(36):e39565. 10.1097/MD.0000000000039565. 39252234 10.1097/MD.0000000000039565 PMC11384829

[4] Rogerson SJ, Mwapasa V, Meshnick SR. Malaria in pregnancy: linking immunity and pathogenesis to prevention. Am J Trop Med Hyg. 2007;77(SUPPL. 6):14–22. 10.4269/ajtmh.77.6.suppl.14. 18165470

[5] Okova D, Lukwa AT, Oyando R, Bodzo P, Chiwire P, Alaba OA. Malaria prevention for pregnant women and under-five children in 10 sub-Saharan Africa countries: socioeconomic and temporal inequality analysis. Int J Environ Res Public Health. 2024;21(12):1–25. 10.3390/ijerph21121656 PMC11675204 39767495

[6] The Global Fund. Investment Case Case: Eighth Replenishment 2025. 2025. https://www.theglobalfund.org/media/t1qnxags/core_2025-investment-case_report_en.pdf. Accessed 1 Aug 2025

[7] Muhammad FM, Majdzadeh R, Nedjat S, Sajadi HS, Parsaeian M. Socioeconomic inequality in intermittent preventive treatment using Sulphadoxine pyrimethamine among pregnant women in Nigeria. BMC Public Health. 2020;20(1):1–9. 33276756 10.1186/s12889-020-09967-w PMC7716500

[8] Ameyaw EK. Individual, community and societal correlates of insecticide treated net use among pregnant women in sub-Saharan Africa: a multi-level analysis. BMC Public Health. 2021;21(1):1–13. 34445978 10.1186/s12889-021-11635-6 PMC8394092

[9] Finda MF, Moshi IR, Monroe A, Limwagu AJ, Nyoni AP, Swai JK, et al. Linking human behaviours and malaria vector biting risk in south-eastern Tanzania. PLoS ONE. 2019;14(6):1–23. 10.1371/journal.pone.0217414 PMC6546273 31158255

[10] Riddle AY, Ssegawa E, Ahumuza S, Meyers L. USAID’s Malaria Action Program for Districts: Gender Analysis. 2017. https://banyanglobal.com/wp-content/uploads/2018/03/MAPD-Gender-Analysis-Report.pdf. Accessed 1 Aug 2025

[11] Mukisa MC, Kassano JJ, Mwalugelo YA, Ntege C, Kahamba NF, Finda MF, et al. Analysis of the 24-h biting patterns and human exposures to malaria vectors in south-eastern Tanzania. Parasit Vectors. 2024;17(1):1–19. 10.1186/s13071-024-06521-0. 39478627 10.1186/s13071-024-06521-0 PMC11526538

[12] Towoliu BI, Sangari F, Permana DE. Questioning the readiness of Manado as a tourism destination: poor service of waitresses in the local restaurants. J Indonesian Tourism Dev Stud. 2017;5(1):9–18. 10.21776/ub.jitode.2017.005.01.02.

[13] Mcclure EM, Goldenberg RL, Dent AE, Meshnick SR. A systematic review of the impact of malaria prevention in pregnancy on low birth weight and maternal anemia. Int J Gynaecol Obstet. 2013;121(2):103–9. 10.1016/j.ijgo.2012.12.014. 23490427 10.1016/j.ijgo.2012.12.014

[14] Alonso S, Chaccour CJ, Elobolobo E, Nacima A, Candrinho B, Saifodine A, et al. The economic burden of malaria on households and the health system in a high transmission district of Mozambique. Malaria J. 2019;18(1):1–10. 10.1186/s12936-019-2995-4. 10.1186/s12936-019-2995-4 PMC6849240 31711489

[15] Ricci F. Social implications of malaria and their relationships with poverty. Mediterr J Hematol Infect Dis. 2012. 10.4084/mjhid.2012.048. 22973492 10.4084/MJHID.2012.048 PMC3435125

[16] Gamble C, Ekwaru PJ, Garner P, Ter Kuil FO. Insecticide-treated nets for the prevention of malaria in pregnancy: a systematic review of randomised controlled trials. PLoS Med. 2007;4(3):506–15. 10.1371/journal.pmed.0040107 PMC1831739 17388668

[17] Onyinyechi OM, Ismail S, Nashriq Mohd Nazan AI. Prevention of malaria in pregnancy through health education intervention programs on insecticide-treated nets use: a systematic review. BMC Public Health. 2024. 10.1186/s12889-024-17650-7. 38468243 10.1186/s12889-024-17650-7 PMC10929229

[18] Yulizawati Y, Silmi H, Intasir MP. Pregnancy-related malaria prevention with insecticide-treated nets (ITNs): a review of the relevant literature. J Midwifery. 2023;8(2):69. 10.25077/jom.8.2.69-75.2023.

[19] WHO. WHO policy brief for the implementation of intermittent preventive treatment of malaria in pregnancy April 2013 (revised January 2014 ). WHO Dep Matern Newborn, Child Adolesc Heal. 2014. http://whqlibdoc.who.int/hq/2001/WHO_RHR_01.30.pdf. Accessed 1 Aug 2025

[20] Apinjoh TO, Anchang-Kimbi JK, Mugri RN, Tangoh DA, Nyingchu RV, Chi HF, et al. The effect of Insecticide Treated Nets (ITNs) on *plasmodium falciparum* infection in rural and semi-urban communities in the South West Region of Cameroon. PLoS ONE. 2015;10(2):1–13. 10.1371/journal.pone.0116300 PMC4340618 25714837

[21] Sumaila I, Issifu S, Asumah MN, Nimo-Boakye K, Awuah DA, Agodzo H, et al. Determinants of insecticide treated bed nets use among pregnant women in the Kintampo north municipality, Bono East region Ghana. medRxiv. 2025. 10.1101/2025.05.13.25327488.

[22] Awunyo W, Agbleta DG, Udeoha MA, Kodjo MM, Afaya A. Ownership and utilization of mosquito bed net among pregnant women in Ghana: a national population-based survey. Trop Med Health. 2025. 10.1186/s41182-025-00739-z. 40346720 10.1186/s41182-025-00739-z PMC12063252

[23] Ladu HI, Shuaibu U, Pulford J. Reasons for mosquito net non-use in malaria-endemic countries: a review of qualitative research published between 2011 and 2021. Trop Med Int Health. 2024;29(7):647–56. 38796689 10.1111/tmi.14006

[24] Terefe B, Habtie A, Chekole B. Insecticide-treated net utilization and associated factors among pregnant women in East Africa: evidence from the recent national demographic and health surveys, 2011–2022. Malar J. 2023;22(1):1–9. 10.1186/s12936-023-04779-w. 37964377 10.1186/s12936-023-04779-w PMC10647126

[25] Demoze L, Adane KC, Gizachew N, Tesfaye AH, Yitageasu G. Utilization of insecticide-treated nets among pregnant women in East Africa: evidence from a systematic review and meta-analysis. BMC Public Health. 2024. 10.1186/s12889-024-20621-7. 39511579 10.1186/s12889-024-20621-7 PMC11545497

[26] World Health Organization. Updates from the Global Malaria Programme. The Global Malaria WHO Malaria Policy Advisory Group (MPAG) meeting report. WHO policy Advis. 2023. 1–22.

[27] Adeniyi L, Chestnutt EG, Rotimi K, Iwegbu A, Oresanya O, Smith J, et al. Delivering insecticide-treated nets (ITNs) through a digitized single-phase door-to-door strategy: lessons from Ondo state, Nigeria. Malar J. 2024. 10.1186/s12936-024-05145-0. 39468541 10.1186/s12936-024-05145-0 PMC11520882

[28] Abu Bonsra E, Osei PA, Sekyi AG, Kyere GA. Insecticide-treated bed nets (ITN) ownership and utilization patterns among caregivers with children under five years: a community-based cross-sectional study in Battor, North Tongu District, Ghana. PLOS Glob Public Heal. 2025;5(2):1–18. 10.1371/journal.pgph.0004228. 10.1371/journal.pgph.0004228 PMC11801732 39913545

[29] Olapeju B, Choiriyyah I, Bertram K, Piccinini D, Harig H, Selby RA, et al. Who buys nets? Factors associated with ownership and use of purchased mosquito nets in sub-Saharan Africa. Malar J. 2019;18(1):1–10. 10.1186/s12936-019-3020-7. 31801579 10.1186/s12936-019-3020-7 PMC6894199

[30] Aguma HB, Rukaari M, Nakamatte R, Achii P, Miti JT, Muhumuza S, et al. Mass distribution campaign of long-lasting insecticidal nets (LLINs) during the COVID-19 pandemic in Uganda: lessons learned. Malar J. 2023;22(1):1–11. 10.1186/s12936-023-04753-6. 37845711 10.1186/s12936-023-04753-6 PMC10577996

[31] Koenker H, Yukich J, Erskine M, Opoku R, Sternberg E, Kilian A. How many mosquito nets are needed to maintain universal coverage: an update. Malar J. 2023;22(1):1–15. 10.1186/s12936-023-04609-z. 37391703 10.1186/s12936-023-04609-z PMC10314435

[32] Shannon J, Kagone M, Candrinho B, Otikwu S, Ingabire C, Gansane A, et al. A qualitative look at bed net access and use in Burkina Faso, Mozambique, Nigeria, and Rwanda following piloted distributions of dual-active ingredient insecticide-treated nets. Malar J. 2024. 10.1186/s12936-024-04868-4. 38715035 10.1186/s12936-024-04868-4 PMC11077758

[33] Balami AD, Said SM, Zulkefli NAM, Norsa’Adah B, Audu B. Knowledge, motivation, self-efficacy, and their association with insecticidal net use among pregnant women in a secondary health centre in Maiduguri, Nigeria. Malar J. 2018. 10.1186/s12936-018-2518-8. 30314438 10.1186/s12936-018-2518-8 PMC6186119

[34] Ankomah A, Adebayo SB, Arogundade ED, Anyanti J, Nwokolo E, Ladipo O, et al. Determinants of insecticide-treated net ownership and utilization among pregnant women in Nigeria. BMC Public Health. 2012;12(1):105. 22309768 10.1186/1471-2458-12-105 PMC3340311

[35] Yaya S, Uthman OA, Amouzou A, Bishwajit G. Mass media exposure and its impact on malaria prevention behaviour among adult women in sub-Saharan Africa: results from malaria indicator surveys. Glob Health Res Policy. 2018;3(1):1–9. 10.1186/s41256-018-0075-x. 29998191 10.1186/s41256-018-0075-x PMC6030754

[36] Tassembedo M, Coulibaly S, Ouedraogo B. Factors associated with the use of insecticide-treated nets: analysis of the 2018 Burkina Faso Malaria Indicator Survey. Malar J. 2021. 10.1186/s12936-021-03756-5. 34001104 10.1186/s12936-021-03756-5 PMC8130301

[37] Ghana Statistical Service (GSS) and ICF. Ghana malaria indicator Survey 2019 [Internet]. National malaria control programme. Accra and Rockville, Maryland. 2020. https://dhsprogram.com/pubs/pdf/MIS35/MIS35.pdf. Accessed 1 Aug 2025

[38] National Malaria Elimination Programme (NMEP), National Population Commission (NPC), ICF. Nigeria Malaria Indicator Survey 2021 Final Report. Abuja, Nigeria, and Rockville, Maryland, USA. 2022. https://dhsprogram.com/pubs/pdf/MIS41/MIS41.pdf. Accessed 1 Aug 2025

[39] Uganda National Malaria Control Division (NMCD), Uganda Bureau of Statistics (UBOS) and I. Uganda Malaria Indicator Survey 2018–19. Kampala, Uganda. 2020. https://www.dhsprogram.com/pubs/pdf/MIS34/MIS34.pdf. Accessed 1 Aug 2025

[40] Leyland AH, Groenewegen PP. What Is Multilevel Modelling? In: Multilevel modelling for public health and health services research: Health in context. Cham: Springer International Publishing; 2020. 33347097

[41] Jensen HAR, Lau CJ, Davidsen M, Feveile HB, Christensen AI, Ekholm O. The impact of non-response weighting in health surveys for estimates on primary health care utilization. Eur J Public Health. 2022;32(3):450–5. 35373254 10.1093/eurpub/ckac032 PMC9159316

[42] Chatrchi G, Duval MC, Brisebois F, Thomas S. The impact of typical survey weighting adjustments on the design effect: a case study special issue. Surv Methods Insights from F. 2015. 10.13094/SMIF-2015-00006.

[43] Osborne A, Bangura C. Predictors of insecticide-treated bed nets use among pregnant women in Sierra Leone: evidence from the 2019 Sierra Leone Demographic Health Survey. Malar J. 2024. 10.1186/s12936-024-05018-6. 38898414 10.1186/s12936-024-05018-6 PMC11188154

[44] Kawuki J, Donkor E, Gatasi G, Nuwabaine L. Mosquito bed net use and associated factors among pregnant women in Rwanda: a nationwide survey. BMC Pregnancy Childbirth. 2023;23(1):1–10. 37280560 10.1186/s12884-023-05583-9 PMC10243234

[45] Yirsaw AN, Gebremariam RB, Getnet WA, Mihret MS. Insecticide-treated net utilization and associated factors among pregnant women and under-five children in East Belessa District, Northwest Ethiopia: using the Health Belief model. Malar J. 2021. 10.1186/s12936-021-03666-6. 33663516 10.1186/s12936-021-03666-6 PMC7971121

[46] Sangaré LR, Weiss NS, Brentlinger PE, Richardson BA, Staedke SG, Kiwuwa MS, et al. Determinants of use of insecticide treated nets for the prevention of malaria in pregnancy: Jinja, Uganda. PLoS ONE. 2012. 10.1371/journal.pone.0039712. 22745817 10.1371/journal.pone.0039712 PMC3382147

[47] Ikeako L, Azuike E, Njelita I, Nwachukwu C, Okafor K, Nwosu C, et al. Insecticide treated nets: perception and practice among pregnant women accessing antenatal services at a tertiary hospital in Awka Nigeria. MOJ Public Heal. 2017;5(4):117–20.

[48] Klu D, Aberese-Ako M, Manyeh AK, Immurana M, Doegah P, Dalaba M, et al. Mixed effect analysis of factors influencing the use of insecticides treated bed nets among pregnant women in Ghana: evidence from the 2019 Malaria Indicator Survey. BMC Pregnancy Childbirth. 2022. 10.1186/s12884-022-04586-2. 35346098 10.1186/s12884-022-04586-2 PMC8958761

[49] World Health Organization (WHO). Malaria in pregnancy: Guidelines for measuring key monitoring and evaluation indicators. France. 2007. https://iris.who.int/bitstream/handle/10665/43700/9789241595636_eng.pdf/iris/bitstream/handle/10665/43700/9789241595636_eng.pdf. Accessed 1 Aug 2025

[50] Kassie GA, Adella GA, Gebrekidan AY, Gebeyehu NA, Gesese MM, Abebe EC, et al. Insecticide-treated bed net utilization and associated factors among pregnant women in Ethiopia: a systematic review and meta-analysis. Malar J. 2023. 10.1186/s12936-023-04655-7. 37533029 10.1186/s12936-023-04655-7 PMC10398969

[51] WHO. World malaria report 2022. Geneva. 2022. https://cdn.who.int/media/docs/default-source/malaria/world-malaria-reports/world-malaria-report-2022.pdf?sfvrsn=40bfc53a_4. Accessed 1 Aug 2025

[52] Mangusho C, Mwebesa E, Izudi J, Aleni M, Dricile R, Ayiasi RM, et al. High prevalence of malaria in pregnancy among women attending antenatal care at a large referral hospital in northwestern Uganda: a cross-sectional study. PLoS ONE. 2023. 10.1371/journal.pone.0283755. 37018283 10.1371/journal.pone.0283755 PMC10075480

[53] Mutagonda RF, Kamuhabwa AAR, Minzi OMS, Massawe SN, Maganda BA, Aklillu E. Malaria prevalence, severity and treatment outcome in relation to day 7 lumefantrine plasma concentration in pregnant women. Malar J. 2016;15(1):1–10. 27177586 10.1186/s12936-016-1327-1 PMC4866074

[54] Oyerogba OP, Adedapo A, Awokson T, Odukogbe AT, Aderinto N. Prevalence of malaria parasitaemia among pregnant women at booking in Nigeria. Health Sci Rep. 2023. 10.1002/hsr2.1337. 37305154 10.1002/hsr2.1337 PMC10256616

[55] Abu Bonsra E, Amankwah Osei P, Adjei Kyeremeh E, Adama S, Sekyi AG, King EF. Factors associated with malaria in pregnancy among women attending ANC clinics in selected districts of the Ashanti Region, Ghana. Malar J. 2025;24(1):8. 10.1186/s12936-025-05244-6. 39799328 10.1186/s12936-025-05244-6 PMC11724469

[56] Yaro JB, Ouedraogo A, Diarra A, Sombié S, Ouedraogo ZA, Nébié I, et al. Risk factors for *Plasmodium falciparum* infection in pregnant women in Burkina Faso: a community-based cross-sectional survey. Malar J. 2021. 10.1186/s12936-021-03896-8. 34488770 10.1186/s12936-021-03896-8 PMC8422625

[57] Oumarou ZM, Lamine MM, Issaka T, Moumouni K, Alkassoum I, Maman D, et al. Infection palustre de la femme enceinte à Niamey au Niger. Pan Afr Med J. 2020;37(365):365. 33796178 10.11604/pamj.2020.37.365.20034 PMC7992404

[58] Arnaldo P, Rovira-Vallbona E, Langa JS, Salvador C, Guetens P, Chiheb D, et al. Uptake of intermittent preventive treatment and pregnancy outcomes: Health facilities and community surveys in Chókwè district, southern Mozambique. Malar J. 2018. 10.1186/s12936-018-2255-z. 29530044 10.1186/s12936-018-2255-z PMC5848514

[59] United States (US) Presidents Malaria Initiative. President’s Malaria Initiative Zimbabwe Malaria Operational Plan FY 2023. 2023. www.pmi.gov. Accessed 1 Aug 2025

[60] Manu G, Boamah-Kaali EA, Febir LG, Ayipah E, Owusu-Agyei S, Asante KP. Low utilization of insecticide-treated bed net among pregnant women in the middle belt of Ghana. Malar Res Treat. 2017. 10.1155/2017/7481210. 28828192 10.1155/2017/7481210 PMC5554553

[61] Taremwa K, Anyamene EL, Nwankwo GI, Agbontale MK, Isiko I. Barriers to effective usage of insecticide-treated mosquito nets (ITNS) among women of reproductive age in Tanzania: a national cross-sectional survey. Malar J. 2025. 10.1186/s12936-025-05417-3. 40676603 10.1186/s12936-025-05417-3 PMC12273030

[62] Mohammed Nonterah SM, Abukari R, Issahaku UK, Mumuni J, Hamidu S, et al. Barriers and determinants of insecticide-treated net utilization for malaria prevention among pregnant women in Tamale Metropolis, Ghana. South Asian J Parasitol. 2024;7(4):411–26.

[63] Bashir SG, Ahmed NI, Abdullahi YB, Abdi YH, Abdi MS, Musa MK. The burden of malaria in East Africa: prevalence, risk factors, and control strategies. Malar J. 2025. 10.1186/s12936-025-05492-6. 40781311 10.1186/s12936-025-05492-6 PMC12335108

[64] Wafula ST, Mendoza H, Nalugya A, Musoke D, Waiswa P. Determinants of uptake of malaria preventive interventions among pregnant women in eastern Uganda. Malar J. 2021;20(1):1–8. 10.1186/s12936-020-03558-1. 33390153 10.1186/s12936-020-03558-1 PMC7780677

[65] Young AJ, Eaton W, Worges M, Hiruy H, Maxwell K, Audu BM, et al. A practical approach for geographic prioritization and targeting of insecticide-treated net distribution campaigns during public health emergencies and in resource-limited settings. Malar J. 2022;21(1):1–13. 10.1186/s12936-021-04028-y. 34983558 10.1186/s12936-021-04028-y PMC8724754

[66] Azanaw J, Worede EA. Determinants of access to insecticide-treated nets in Sub-Saharan Africa: a multilevel cross-country analysis using 29 DHS data. PLoS ONE. 2025;20:1–16. 10.1371/journal.pone.0330431. 10.1371/journal.pone.0330431 PMC12422452 40929023

[67] Dun-Dery F, Kuunibe N, Meissner P, Winkler V, Jahn A, Müller O. Determinants of the use of insecticide-treated mosquito nets in pregnant women: a mixed-methods study in Ghana. Int Health. 2022;14(6):619–31. 35064966 10.1093/inthealth/ihab087 PMC9623492

[68] Adedokun ST, Uthman OA. Individual and contextual correlates of mosquito net use among women in Nigeria. Malar J. 2020;19(1):1–10. 32264875 10.1186/s12936-020-03219-3 PMC7137195

[69] Getnet Y, Teym A, Wubie M, Shiferaw S, Tilahun Assaye B, Aneley Z, et al. Long lasting insecticide-treated nets utilization and associated factors among pregnant women in Shebel Berenta District, Northwest Ethiopia. Environ Health Insights. 2024. 10.1177/11786302241291957. 39494047 10.1177/11786302241291957 PMC11528622

[70] Quisumbing AR, Meinzen-dick R, Raney TL, Croppenstedt A, Behrman JA, Peterman A. Why close gender gaps. Interational food policy Res Inst. 2014. https://www.aesanetwork.org/wp-content/uploads/2018/02/Gender-in-Agriculture-Closing-the-Knowledge-Gap.pdf. Accessed 1 Aug 2025

[71] Bradshaw S, Chant S, Linneker B. Gender and poverty: what we know, don’t know, and need to know for Agenda 2030. Gend Place Cult. 2017;24(12):1667–88. 10.1080/0966369X.2017.1395821.

[72] Eshetu B, Bekele H, Debella A, Eyeberu A, Balis B, Habte S, et al. Insecticide-treated net utilization and associated factors among pregnant women in Ethiopia: a systematic review and meta-analysis. Front Glob Womens Health. 2023;4:1–12. 10.3389/fgwh.2023.1147583. 10.3389/fgwh.2023.1147583 PMC10657856 38025984

[73] Seyoum TF, Andualem Z, Yalew HF. Insecticide-treated bed net use and associated factors among households having under-five children in East Africa: a multilevel binary logistic regression analysis. Malar J. 2023;22(1):1–9. 10.1186/s12936-022-04416-y. 36611186 10.1186/s12936-022-04416-y PMC9826573

